# DDR2 signaling and mechanosensing orchestrate neuroblastoma cell fate through different transcriptome mechanisms

**DOI:** 10.1002/2211-5463.13798

**Published:** 2024-03-27

**Authors:** Theadora Vessella, Steven Xiang, Cong Xiao, Madelyn Stilwell, Jaidyn Fok, Jason Shohet, Esteban Rozen, H. Susan Zhou, Qi Wen

**Affiliations:** ^1^ Department of Chemical Engineering Worcester Polytechnic Institute MA USA; ^2^ Bancroft School Worcester MA USA; ^3^ Nash Family Department of Neuroscience, Friedman Brain Institute Icahn School of Medicine at Mount Sinai New York NY USA; ^4^ Black Family Stem Cell Institute Icahn School of Medicine at Mount Sinai New York NY USA; ^5^ Department of Biomedical Engineering Wichita State University KS USA; ^6^ Department of Neurobiology University of Massachusetts Medical School Worcester MA USA; ^7^ Department of Pediatrics University of Massachusetts Medical School Worcester MA USA; ^8^ Crnic Institute Boulder Branch, BioFrontiers Institute University of Colorado Boulder CO USA; ^9^ Department of Physics Worcester Polytechnic Institute MA USA

**Keywords:** DDR2, ECM stiffness, pro‐proliferation, RNA‐seq, senescence, transcriptome

## Abstract

The extracellular matrix (ECM) regulates carcinogenesis by interacting with cancer cells via cell surface receptors. Discoidin Domain Receptor 2 (DDR2) is a collagen‐activated receptor implicated in cell survival, growth, and differentiation. Dysregulated DDR2 expression has been identified in various cancer types, making it as a promising therapeutic target. Additionally, cancer cells exhibit mechanosensing abilities, detecting changes in ECM stiffness, which is particularly important for carcinogenesis given the observed ECM stiffening in numerous cancer types. Despite these, whether collagen‐activated DDR2 signaling and ECM stiffness‐induced mechanosensing exert similar effects on cancer cell behavior and whether they operate through analogous mechanisms remain elusive. To address these questions, we performed bulk RNA sequencing (RNA‐seq) on human SH‐SY5Y neuroblastoma cells cultured on collagen‐coated substrates. Our results show that DDR2 downregulation induces significant changes in the cell transcriptome, with changes in expression of 15% of the genome, specifically affecting the genes associated with cell division and differentiation. We validated the RNA‐seq results by showing that DDR2 knockdown redirects the cell fate from proliferation to senescence. Like DDR2 knockdown, increasing substrate stiffness diminishes cell proliferation. Surprisingly, RNA‐seq indicates that substrate stiffness has no detectable effect on the transcriptome. Furthermore, DDR2 knockdown influences cellular responses to substrate stiffness changes, highlighting a crosstalk between these two ECM‐induced signaling pathways. Based on our results, we propose that the ECM could activate DDR2 signaling and mechanosensing in cancer cells to orchestrate their cell fate through distinct mechanisms, with or without involving gene expression, thus providing novel mechanistic insights into cancer progression.

## Graphical abstract

The ECM chemical composition and mechanical properties are crucial for tumor development. This study demonstrates that both knocking down the collagen receptor DDR2 and increasing ECM stiffness can reduce neuroblastoma cell proliferation. Surprisingly, RNA sequencing results show that the DDR2 knockdown significantly enhances the expression tumor suppressor genes in neuroblastoma cells, while the ECM stiffness does not.

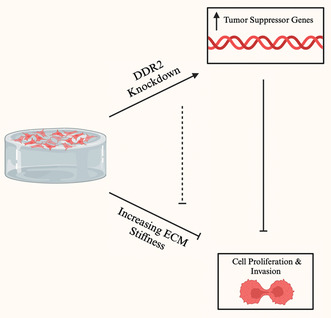

**Abbreviations**
DDR2: Discoidin Domain Receptor 2
DEG: differentially expressed gene
DMEM: Dulbecco's modified Eagle's medium
ECM: extracellular matrix
GO: gene ontology
IGF2: insulin growth factor 2
KEGG: Kyoto encyclopedia of genes and genomes
PAA: polyacrylamide
PCA: principal component analysis
PLK1: polo‐like kinase 1
PTEN: phosphatase and tensin homolog
RNA‐seq: RNA sequencing
TERT: telomerase reverse transcriptase

A hallmark of cancer cells that distinguishes them from normal differentiated cells is their unrestricted proliferative ability. Numerous genetic, signaling, and metabolic pathways have been implicated in cancer proliferation. Broadly, these factors are divided into pro‐proliferative and anti‐proliferative ones [1]. The former includes those that promote DNA and protein synthesis including PI3 kinase and Ras‐MAPK signaling, chromosome segregation, and telomere extension just to name a few, whereas the latter acts as a brake for proliferation including the tumor suppressors PTEN and p53 [2, 3]. Genetic mutation of both positive and negative regulators has been causally linked to various cancer types. Not surprisingly, hampering cancer proliferation is a primary target of cancer drugs, for example, paclitaxel (Taxol), the most prescribed drug for treating cancers, acts by inhibiting microtubule assembly to prevent cell division [4]. Despite extensive studies, however, how cancer cells could escape from cell division regulation is not fully understood.

Cancer cells grow in a microenvironment wherein they closely interact with the extracellular matrix (ECM). As a major ECM component, collagen composition regulates various steps of cancer progression including growth, invasion, and metastasis, partly through activation of its canonical receptor integrin to regulate cytoskeleton organization and cell motility [5, 6, 7]. Recently, discoidin domain receptor tyrosine kinase 2 (DDR2), a non‐typical collagen receptor that is dysregulated in various cancer types, has emerged as a key signaling molecule in carcinogenesis [8, 9]. Collagen binding to DDR2 activates its tyrosine kinase activity to initiate canonical pathways such as ERK/MAPK and PI3K/AKT signaling cascades [10, 11, 12]. Despite these studies, how DDR2 regulates cancer cell behavior is incompletely understood.

Besides providing biochemical cues that elicit signaling in cancer cells, ECM components also establish the biomechanical environment that critically controls cancer progression [13, 14]. Upregulated collagen production and altered collagen fiber organization result in stiffening of the tumor ECM environment. Such increased tissue stiffness has been exploited as a marker for detection of solid tumors [15]. Integrin‐mediated mechanotransduction has been proposed to regulate ECM stiffness‐dependent signaling pathways that play pivotal roles in cell growth, proliferation, and survival [16, 17]. High ECM stiffness has been reported to facilitate cancer metastasis by triggering epithelial‐mesenchymal transition [17, 18], promoting cancer cell proliferation, and boosting resistance to chemotherapy [19]. A recent study further suggested that DDR2 regulates the integrin‐mediated mechanotransduction functions of cancer‐associated fibroblasts [20]. However, whether DDR2 and ECM biomechanics could interact to regulate cancer cell behavior is yet to be determined.

To systematically examine the effects of DDR2 signaling and substrate stiffness on cancer cells, in the present study, we performed RNA‐seq analysis of a human neuroblastoma cell line SH‐SY5Y. This cell line has been extensively used as a model to study cancer progression [21]. We found that shRNA knockdown of DDR2 alters global gene expression of SH‐SY5Y cells and inhibits cell proliferation. Moreover, we found that increasing substrate stiffness also slows down proliferation, similar to DDR2 knockdown. However, RNA‐seq revealed no gene expression changes associated with increasing substrate stiffness. These data suggest that DDR2 signaling and biomechanics, two downstream effectors of ECM, could regulate cancer cell proliferation through different mechanisms, with or without involvement of gene expression.

## Results

### Bulk RNA‐seq revealed a profound impact of DDR2 on transcriptome

**Fig. 1:**
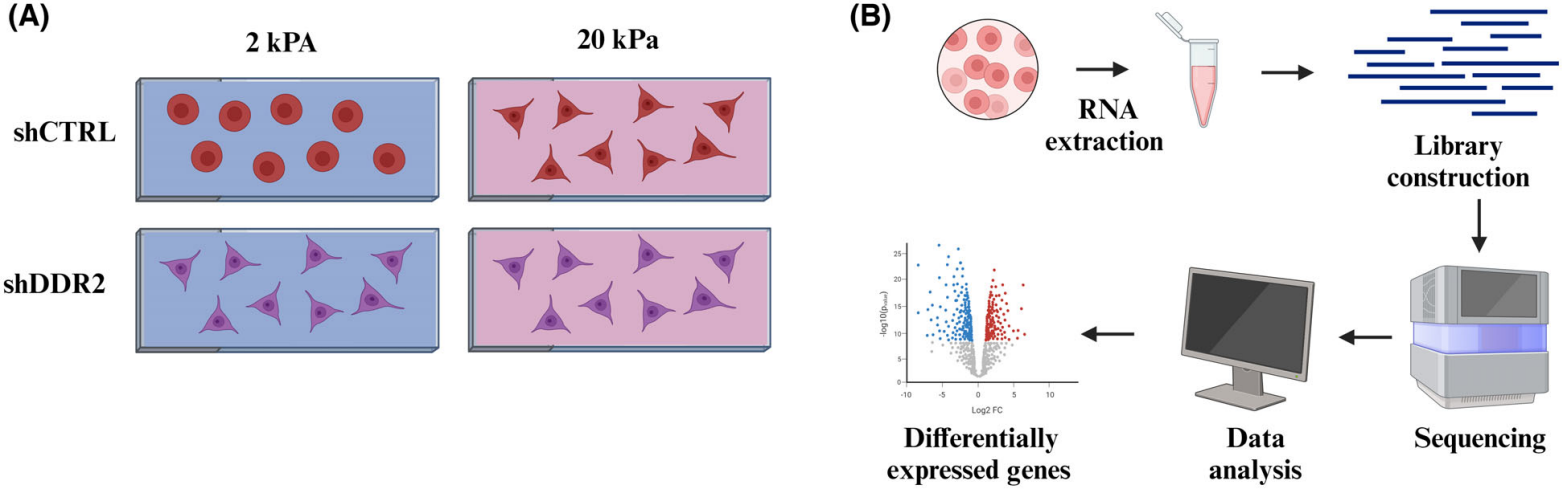
Schematic illustration of experimental process to find novel genes involved in biophysical stimulation. (A) The four sample conditions used in this study: shCTRL and shDDR2 cells cultured on 2 and 20 kPa collagen‐coated polyacrylamide gel substrates. (B) Experimental process of RNA‐seq: a 24‐h incubation of the cells on PAA gels followed by cellular RNA extraction, library construction, sequencing, and data analysis. This figure is created with BioRender.com.

**Fig. 2:**
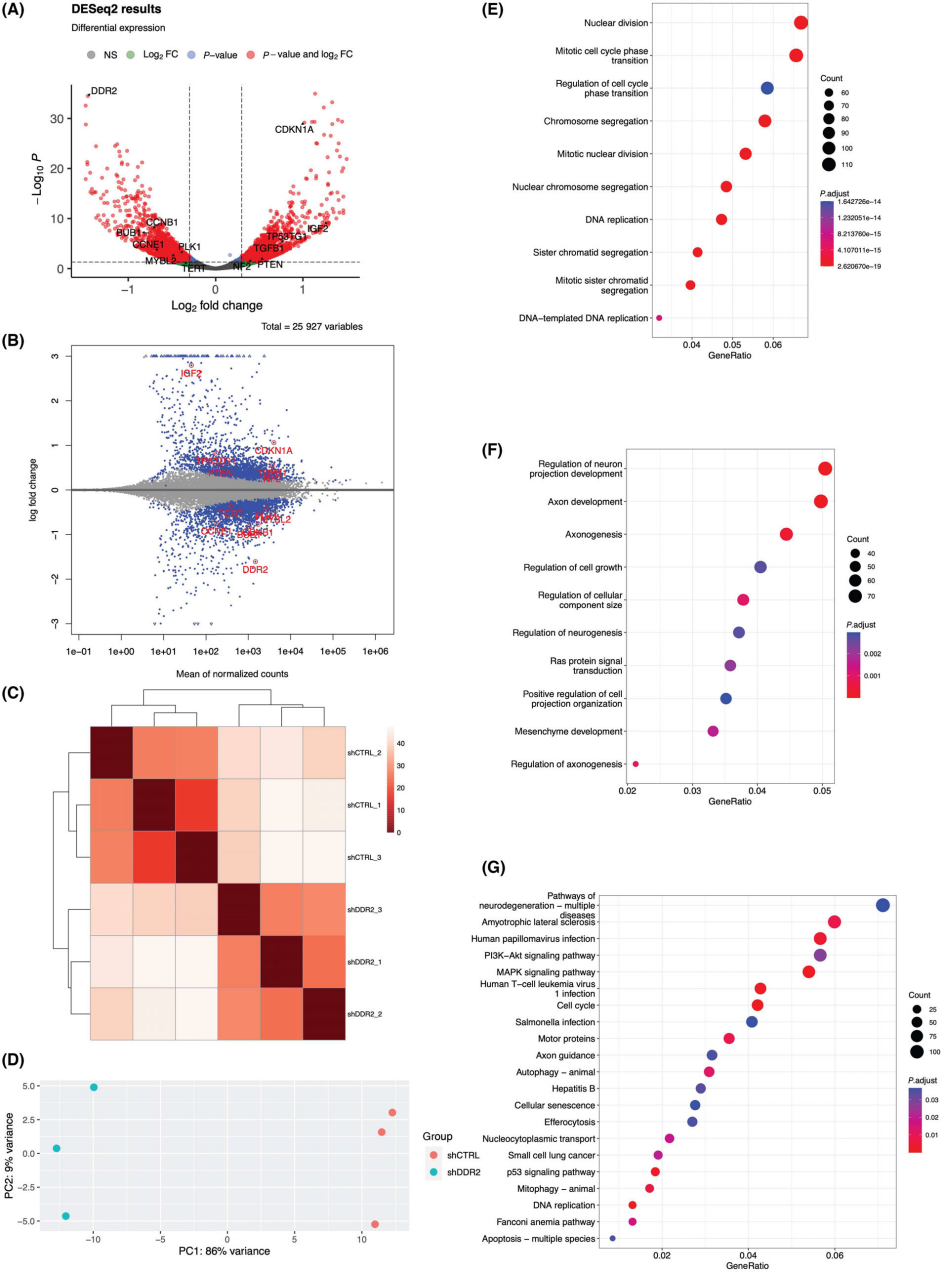
RNA‐seq data analysis of shCTRL vs shDDR2 cells on soft substrates. (A) Volcano plot of RNA‐seq data in the malignant neuroblastoma cell line SH‐SY5Y where the *x*‐axis represents fold change in transcripts from shCTRL vs shDDR2 cell lines (a positive score represents enrichment; a negative score represents depletion). The *y*‐axis represents statistical confidence for each *x*‐axis point. (B) MA plot of RNA‐seq data, where the *x*‐axis represents statistical confidence for each *y*‐axis point. The *y*‐axis represents fold change in transcripts from shCTRL vs shDDR2 cell lines. (C) Heatmap analysis of relationships among different samples. (D) PCA analysis of sample clustering. (E) GO biological process analysis of the down differentially expressed genes. (F) GO biological process analysis of the up differentially expressed genes. (G) KEGG enrichment analysis of the differentially expressed genes.

To survey the effects of DDR2 knockdown on SH‐SY5Y cells, we compared shCTRL and shDDR2 cells grown on collagen‐coated 2 kPa Polyacrylamide (PAA) gels. SH‐SY5Y cells were stably transduced with a lentiviral shRNA construct to target DDR2 expression (shDDR2 SH‐SY5Y) or a non‐targeting vector (shCTRL SH‐SY5Y; Fig. 1A, see Materials and methods). We harvested the cells cultured on the PAA gels 24 h after plating, extracted RNA, constructed libraries, did the sequencing, and analyzed the RNA‐seq data (Fig. 1B). Three biological repeats were conducted for each sample. Volcano plot and MA plot analysis of the total 3923 differentially expressed genes (DEGs) revealed that 1982 genes reduced their expression in shDDR2 cells when compared to shCTRL, whereas another 1941 genes upregulated their expression (Fig. 2A,B, Table S2). As expected, DDR2 was among the most significant reduced genes, with a 63% reduction in shDDR2 cells when compared to shCTRL cells with a *P*‐adj value of 3.3 × 10^−35^ (Fig. 2A, Fig. S1A). Reduction of the DDR2 mRNA level was further confirmed by quantitative PCR (Fig. S1B). Heat map and principal component analysis (PCA) revealed that shDDR2 and shCTRL groups were well segregated (Fig. 2C,D). With the number of DEGs representing ~ 15% of the entire human genome, our results demonstrate a profound role of DDR2 in regulation of SH‐SY5Y cell transcriptome.

### Categorization and GO analysis

We performed gene ontology (GO) analysis of DEGs in shDDR2 SH‐SY5Y cells. Strikingly, the GO biological process analysis showed that those downregulated genes were enriched in the pathways related to cell proliferation, including nuclear division, mitotic cell cycle phase transition, chromosome segregation, DNA replication, and sister chromatid segregation, just to name a few (Fig. 2E, Table S4). Our analysis suggested that a normal function of DDR2 is to maintain fast proliferation of SH‐SY5Y cells. When DDR2 is knocked down, proliferation of SH‐SY5Y cells is expected to be reduced.

Next, we performed GO biological process analysis of those upregulated DEGs in shDDR2 SH‐SY5Y cells. Intriguingly, we found the top pathways were related to regulation of cell growth and regulation of cellular component size (Fig. 2F, Table S5), suggestive of stimulation of cellular growth and size after DDR2 knockdown. In addition, we noted that genes involved in neuronal development such as regulation of neurogenesis, axonogenesis, and regulation of neuron projection development were also upregulated (Fig. 2F), suggesting differentiation of SH‐SY5Y cells towards a neuronal fate after DDR2 knockdown. Indeed, SH‐SY5Y cells are a neuroblastoma line and could be readily induced into dopaminergic neurons upon proper stimulation [21].

We further continued the pathway analysis by examining all DEGs, including up‐ and downregulated genes using the Kyoto encyclopedia of genes and genomes (KEGG) database. One pathway showing significant gene enrichment was related to cell senescence, which is known as a viable but non‐proliferation cell state (Fig. 2G). Collectively, our GO and KEGG analyses support the notion that a normal function of DDR2 would be to maintain a fast proliferative, cancerous state of SH‐SY5Y cells by preventing their differentiation.

### Identification of a gene cohort controlling proliferation, senescence, and cell size

The proliferative signature of cancer cells is known to be causally linked to expression of a core set of pro‐proliferative genes including *MYBL2*, *BUB1*, and *polo‐like kinase 1* (*PLK1*) [22]. In addition, genes that promote cell cycle progression such as *CCNE1* and *CCNB1*, which encode cyclin E1 and cyclin B1, respectively, are also crucial players in cancer cell proliferation [22]. Remarkably, we found a reduction of all these positive regulation genes of cell proliferation in shDDR2 SH‐SY5Y cells (Fig. 2A,B, Fig. S2A).

As mentioned above, cell proliferation is also subject to negative regulation. For example, phosphatase and tensin homolog (*PTEN*) is one of best‐known tumor suppressors. In human tumors, *PTEN* expression is usually suppressed through promoter methylation, allowing cancer cells to proliferate unrestrictedly. As expected, we found that *PTEN* expression was significantly increased after DDR2 knockdown (Fig. S2B). PTEN is a negative regulator of PI3K‐Akt signaling [23]. Consistently, we found reduced expression of *PIK3CA* that encodes a PI3K subunit (Table S2). Furthermore, expression of *NF2*, another tumor suppressor [24], was increased after DDR2 RNAi (Fig. S2B). These data suggested that DDR2 knockdown could reduce the proliferative competence. Along this line. *TGF‐β* is known for its anti‐proliferative effects [25], and expression of *TGF‐β* and genes implicated in TGF‐β signaling was increased after DDR2 knockdown (Fig. 2A,B, Fig. S2B, Table S5).

After each cell division, the length of telomere is reduced and such reduction is thought to limit or prevent normal cells from unlimited proliferation [26]. In normal cells, expression of an enzyme known to increase the telomere length, *telomerase reverse transcriptase* (*TERT*), is largely suppressed. However, cancer cells are unique in that they could upregulate *TERT* expression to ensure telomere length increases after each cell division [26]. We noted that *TERT* expression was significantly reduced after DDR2 knockdown (Fig. S2C). Such *TERT* reduction has been linked to cancer senescence [27]. Supporting the notion that shDDR2 SH‐SY5Y cells are poised to enter senescence, a signature senescence gene, *CDKN1A* [28], was upregulated after DDR2 knockdown (Fig. S2C). Moreover, increasing cell growth and size is often considered as a morphological marker of cell senescence. Indeed, we found that *insulin growth factor 2* (*IGF2*), which is known to stimulate cell growth [29], was increased after DDR2 knockdown (Fig. 2A,B, Fig. S2C).

We next performed quantitative PCR in shCTRL and shDDR2 cells to confirm the RNA‐seq results. We validated mRNA expression changes of 8 out of 11 differentially expressed genes described above (Fig. S3). Two genes, *BUB1* and *PLK1* showed consistent expression changes but did not reach significance, while the third gene *IGF2* failed in our detection (Fig. S3).

### Bulk RNA‐seq revealed no transcriptome changes in response to substrate stiffness

**Fig. 3:**
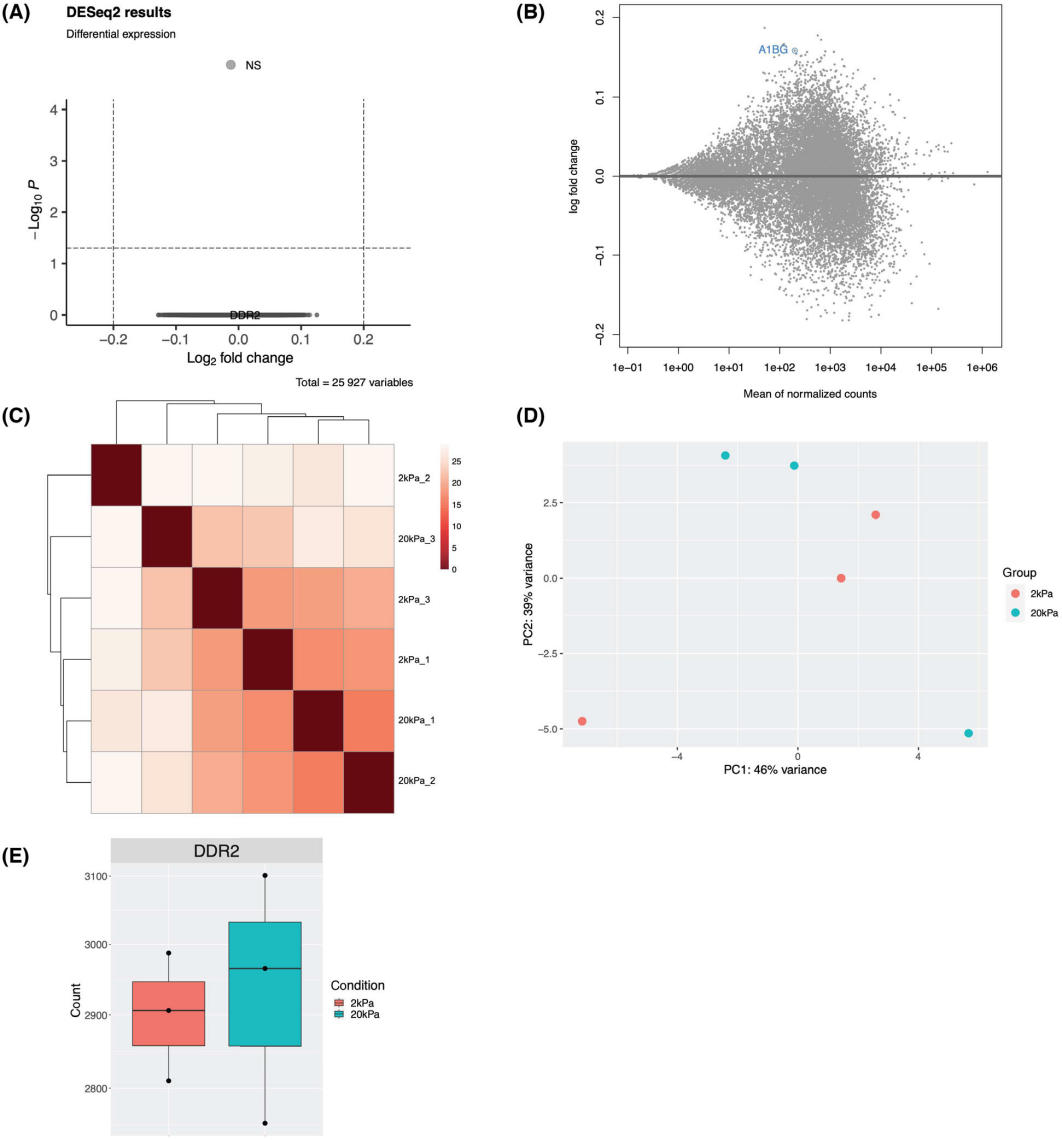
RNA‐seq data analysis of shCTRL cells cultured on hard vs soft substrates. (A) Volcano plot of RNA‐seq data in the malignant neuroblastoma cell line SH‐SY5Y, where the *x*‐axis represents fold change in transcripts from shCTRL cells cultured on hard vs soft substrates (a positive score represents enrichment, a negative score represents depletion). The *y*‐axis represents statistical confidence for each *x*‐axis point. (B) MA plot of RNA‐seq data, where the *x*‐axis represents statistical confidence for each *y*‐axis point. The *y*‐axis represents fold change in transcripts from shCTRL cell lines cultured on hard vs soft substrates. (C) Heatmap analysis of relationships among different samples. (D) PCA analysis of sample clustering. (E) The normalized reads count of shDDR2 in the shCTRL cell line cultured on hard vs soft substrates, *P*adj value 0.999. Each dot represents a biological replicate.

Cancer progression is often associated with changes in their biomechanical properties [14]. Indeed, hardness is often the first sign of tumorigenesis. Neuroblastoma, among many cancer cell types closely interacts with ECM and the crosstalk between cancer and biomechanical cues actively regulates almost all steps of cancer progression [13, 30]. Despite the close interactions between cancer cells and the surrounding biomechanical cues, the impact of biomechanics on cancer cell transcriptomes is yet unknown. Bone marrow and liver, two major metastatic sites of neuroblastoma, exhibit a broad difference of stiffness ranging from hundreds to thousands of Pascal [31, 32, 33]. To assess whether substrate stiffness could regulate gene expression of neuroblastoma cells, we performed bulk RNA‐seq to profile SH‐SY5Y cells grown on 2 and 20 kPa substrate stiffness. Surprisingly, we found no statistically significant changes in any gene expression across the transcriptome (Fig. 3A,B,E). Heatmap and PCA analysis showed that these two groups cannot be separated (Fig. 3C,D). We also explored DEGs in shDDR2 SH‐SY5Y cells grown on 2 and 20 kPa substrate stiffness and found no statistically significant changes in gene expression (data not shown). Nevertheless, we did notice that DEGs associated with DDR2 knockdown were different when cells were cultured at 2 kPa (3923 DEGs) versus 20 kPa (1013 DEGs) (Tables S2 and S3).

### The effects of DDR2 knockdown and substrate stiffness on cell proliferation and senescence

**Fig. 4:**
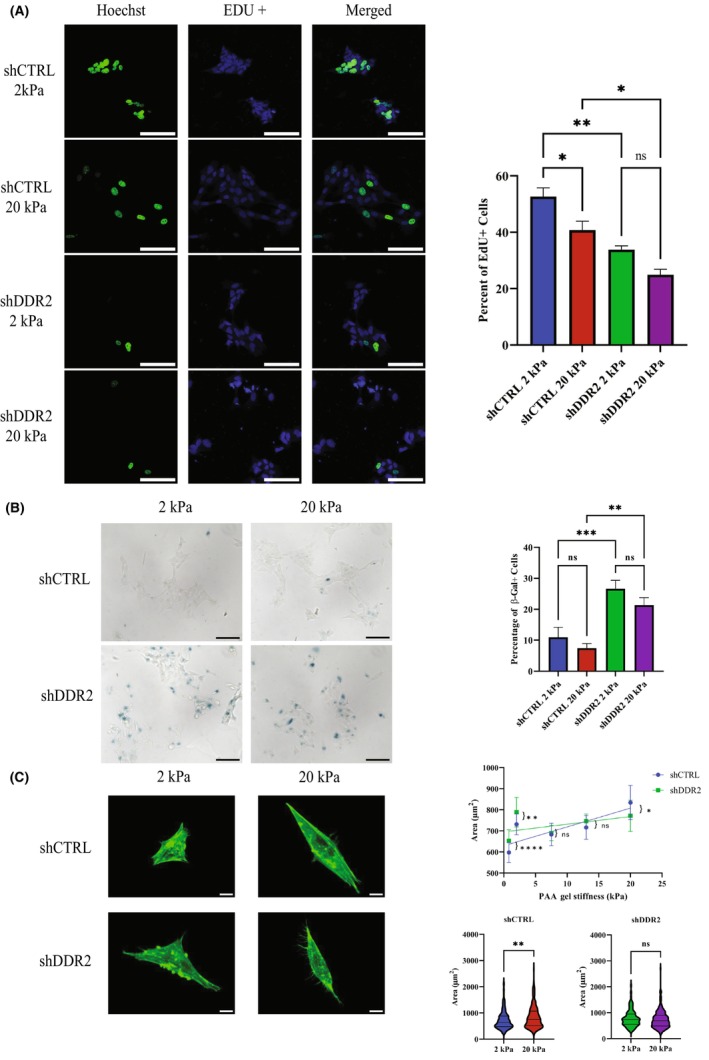
DDR2 knockdown leads to senescence and increased cell area in shDDR2 neuroblastoma cells. (A) Representative images of EdU assay on shCTRL and shDDR2 cells for both soft and hard gels. Blue channel is Hoechst 33342 and green is the positive EdU signal. Percentage of positive EdU cells taken as ratio of EdU positive cells/total number of cells (*n* = 6–15 regions). One‐way ANOVA followed by Bonferroni post‐hoc test, not significant (ns) *P* > 0.05, *P ≤ 0.05, **P ≤ 0.01, ***P ≤ 0.001. Scale bars represent 100 μm. (B) Senescence analysis of shCTRL and shDDR2 cell lines using beta‐galactosidase stain after 48 h in culture. Blue arrow represents a positive beta‐galactosidase signal. One‐way ANOVA followed by Bonferroni *post‐hoc* test, not significant (ns) *P* > 0.05, *P ≤ 0.05, **P ≤ 0.01, ***P ≤ 0.001. Percentage of positive beta‐galactosidase cells taken as ratio of beta‐galactosidase positive cells/total number of cells (*n* = 10 regions). Scale bars represent 100 μm. (C) Representation of cell areas for shCTRL and shDDR2 cells on soft and hard PAA gel (*N* = 3–4, *n* = 224–261 cells). Line graph represents the mean area as a function of substrate stiffnesses (0.8, 2, 7.5, 13, and 20 kPa) based on Mann–Whitney Test, not significant (ns) *P* > 0.05, *P ≤ 0.05, **P ≤ 0.01, ***P ≤ 0.001, ****P ≤ 0.0001. Scale bars represent 10 μm. All error bars represent SEM.

Our RNA‐seq data predicted a reduction of cell proliferation after DDR2 knockdown. To directly test this idea, we performed the EdU cell proliferation assay. EdU as an analog of thymidine is incorporated into DNA selectively in dividing cells. We found that when grown on substrates of either 2 or 20 kPa, EdU labeling was significantly reduced in shDDR2 when compared to shCTRL SH‐SY5Y cells (Fig. 4A). These results provide direct evidence of reduced proliferation after DDR2 knockdown, thus validating our RNA‐seq results.

We also observed a significant reduction in EdU incorporation in shCTRL SHY‐SY5Y cells when substrate stiffness was increased from 2 to 20 kPa (Fig. 4A). This suggests that an increase in substrate stiffness could reduce SH‐SY5Y cell proliferation, despite our RNA‐seq analysis showing no associated changes in gene expression (Fig. 3A,B). On the other hand, increasing of substrate stiffness failed to alter the proliferation of the shDDR2 SH‐SY5Y cells (Fig. 4A), which had reduced DDR2 expression. These results suggest that DDR2 plays an important role in regulating cellular response to substrate stiffness.

Cell senescence is a common outcome of cancer cells that exit cell cycles [34]. To measure cell senescence, we stained for the enzymatic activity of β‐galactosidase, a marker of cell senescence [35]. We found that β‐galactosidase signals were significantly increased in shDDR2 SH‐SY5Y cells when compared to shCTRL SH‐SY5Y cells, when cultured at either 2 or 20 kPa stiffness (Fig. 4B). This result suggests that DDR2 knockdown induces senescence of SH‐SY5Y cells, which is consistent with our RNA‐seq analysis (Fig. 2G). On the other hand, the change in substrate stiffness did not result in significant changes in the β‐galactosidase signals in either shCTRL or shDDR2 SH‐SY5Y cells (Fig. 4B).

The size of cancer cells are related to their states. For example, increased cell sizes are often associated with senescence [36, 37, 38]. We therefore measured the spreading areas of shCTRL cells and shDDR2 cells cultured on substrates of varying stiffness (from 0.8 to 20 kPa). We found that at lower stiffnesses (0.8–2 kPa), shDDR2 cells exhibited larger areas than shCTRL cells (Fig. 4C), consistent with cells entering a senescence state. Moreover, shCTRL cells increased their spreading area when substrate stiffness was increased (Fig. 4C, Fig. S4). However, shDDR2 cells did not change their sizes when cultured on harder substrate (Fig. 4C, Fig. S4), suggesting that DDR2 is indispensable for SH‐SY5Y cells to respond to stiffness changes.

Taken together, our results indicate that downregulation of DDR2 in neuroblastoma cells decreases cell proliferation and induces senescence that is independent of substrate stiffness.

### Effects of DDR2 knockdown on cell contractility

**Fig. 5:**
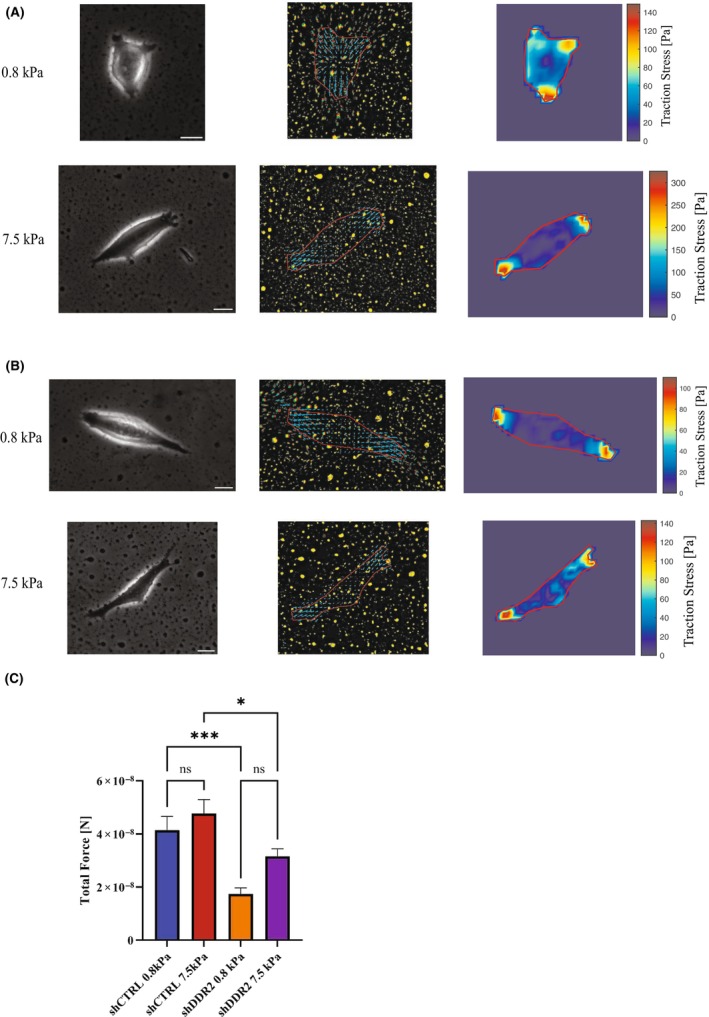
Traction force microscopy on varying stiffness of collagen‐coated PAA gels for shCTRL and shDDR2 cell lines. Representative phase contrast images, bead displacement maps, and stress heat maps for (A) shCTRL and (B) shDDR2 cell lines on 0.8 and 7.5 kPa collagen‐coated PAA gels. Results summary of (C) traction forces of shCTRL and shDDR2 on 0.8 kPa and 7.5 kPa substrate stiffnesses (*n* = 26–29 cells). Error bars represent SEM. Scale bars represent 10 μm. One‐way ANOVA with Bonferroni post‐hoc test, not significant (ns) *P* > 0.05, *P ≤ 0.05, **P ≤ 0.01, ***P ≤ 0.001.

Tumor cells generate force to remodel the ECM and facilitate metastasis. Elevated cellular traction force has previously been shown to correlate with increasing metastatic potential of cancer cells [39, 40, 41]. To investigate whether cells in the senescence state versus cells in the fast proliferative state could exhibit different traction forces, we measured the traction forces of shCTRL and shDDR2 cells cultured on PAA gels. We found that, either on soft substrates (0.8 kPa) or on hard substrates (7.5 kPa), shCTRL cells exhibited a greater total force than shDDR2 cells (Fig. 5A,C). These results underline the importance of DDR2 in cell contractility and suggest that SH‐SY5Y neuroblastoma cells in the fast‐proliferating state are more metastatic than those in the senescent state. In contrast to other cancers such as breast cancer cells that exert stronger traction force on stiffer substrates [42], the SH‐SY5Y neuroblastoma cells do not alter their traction forces in response to varying substrate stiffness (Fig. 5C), revealing a cell type specificity.

## Discussion

The biomechanical properties of ECM change dynamically throughout cancer progression. The stiffened tumor ECM and abnormal mechanosensitivity of cancer cells have been shown to promote metastasis [43, 44, 45]. The ECM receptors on cell surface are key components for transduction of these biochemical cues into intracellular signals. As a non‐typical collagen receptor, DDR2 binds to fibrous collagen I. Dysregulated DDR2 expression has been documented in various cancer types including neuroblastoma. However, how neuroblastoma cells respond to substrate stiffness, as well as the role of DDR2 in sensing substrate stiffness are still poorly understood.

In the present study, we provide to our knowledge the first RNA‐seq study to comprehensively measure the effects of substrate stiffness on neuroblastoma cells. The stiffness of our substrates overlap the stiffness range of the original tissue and metastasis sites of neuroblastoma [32, 33, 46, 47]. We found no changes in gene expression between SH‐SY5Y cells grown on a soft 2 kPa versus a hard 20 kPa substrate. This result is rather unexpected given that our results, along with a few previous studies on neuroblastomas cells, have observed overt changes in cell proliferation, cell size, and cell differentiation in response to stiffness changes [47, 48, 49]. On the other hand, knocking down the collagen receptor DDR2 profoundly altered SH‐SY5Y cell transcriptome. Pathway analysis revealed a downregulation of pro‐proliferative genes and an upregulation of tumor suppressor genes. These predictions were later validated by EdU labeling, β‐galactosidase staining, and cell morphology assays.

Although the roles of DDR2 in the regulation of cell homeostasis, migration or cell cycle remain controversial, a growing number of reports have implicated DDR2 as a key driver of cell proliferation in fibroblasts [50, 51], chondrocytes [50, 52, 53], hepatic stellate cells [54], lung squamous cancer cell lines [55], osteoblasts [53], and breast cancer cell lines [56]. This is consistent with our findings that DDR2 is also required for proper neuroblastoma cell proliferation. Furthermore, a recent study from Xu *et al*. [57] suggested that epigenetic downregulation of DDR2 in human‐derived bone marrow mesenchymal stem cells is associated with reduced proliferation and increased senescence of these cells, which is in agreement with our findings in SH‐SY5Y neuroblastoma cells.

The overlapped cellular responses between DDR2 knockdown and increasing of substrate stiffness, such as proliferation arrest, nevertheless suggest that genetic factors and biomechanical cues could elicit similar cellular responses in SH‐SY5Y cells through distinct mechanisms, with or without involvement of gene expression. What could be the possible cellular responses activated by substrate stiffness to regulate SH‐SY5Y cell proliferation? As metabolism of cancer cells directly control their proliferation [58], one possibility would be that mechanosensing of substrate stiffness could alter cellular metabolism. On the other hand, DDR2 knockdown will alter transcriptome, resulting in similar metabolic reprograming as varying the substrate stiffness. Future studies are needed to test this idea.

shDDR2 SH‐SY5Y cells lost their responses to substrate stiffness as revealed by the EdU and cell area assays. These results indicate that DDR2 could modulate how substrate stiffness influences the SH‐SY5Y cell behavior. To detect substrate stiffness, cells need to exert traction force to deform the substrate, sense the subsequent restoring force, and convert this force signal into biological cues, which activate downstream signaling pathways that control cell morphology, mechanics, migration, and proliferation [14]. Given that some signaling pathways such as MAPK and PI3K‐AKT are regulated by both DDR2 and substrate stiffness [59], it is plausible that DDR2 modulates the responses of cells to substrate stiffness by regulating these downstream pathways. Alternatively, as integrin is implicated in mechanosensing and DDR2 and integrin bind to different sites of fibrillar collagen [60, 61], it is possible that DDR2 could modulate integrin‐mediated mecahnosensing. Consistent with this notion, a recent study on cancer‐associated fibroblasts show that DDR2 activation is required for full activation of *β*1 integrins [20]. Finally, DDR2 depletion has been shown to result in smaller focal adhesions and traction force [20, 48]. In corroboration with this, we observed that DDR2 knockdown resulted in weaker traction forces, suggesting yet another possibility that DDR2 could control the effects of substrate stiffness on SH‐SY5Y cells through modulation of the traction force.

## Materials and methods

### Cell culture

Human neuroblastoma cell line SH‐SY5Y (ATCC, Cat. No. CRL‐2266) stably transduced with the Tet‐pLKO‐puro lentiviral vector expressing either a control non‐targeting shRNA or shDDR2 were kindly provided by Dr. Jason Shohet (UMass Chan Medical School). Cells were cultured in Dulbecco's modified Eagle's medium (DMEM) (Themo Fisher Sci, Eugene, OR, USA, Cat. No. 11960069) supplemented with 10% fetal bovine serum (Gibco, Grand Island, NY, USA, Cat. No. A5670701), 2 mm glutamine (Gibco, Cat. No. 35050061) and antibiotics (penicillin and streptomycin) (Sigma‐Aldrich, St. Louis, MO, USA, Cat. No. P4333).

### Polyacrylamide substrate preparation

Polyacrylamide gel substrates were prepared through the polymerization of acrylamide (Bio‐Rad, Waltham, MA, USA, Cat. No. 1610140) and bis‐acrylamide, with varying concentrations to achieve the desired stiffness levels (Table S1). This polymerization process was initiated by a solution containing 0.1% ammonium persulfate (Amresco, Solon, OH, USA, Cat. No. 7727540) and 0.3% N,N,N′,N′‐tetramethylethylenediamine (Amresco, Cat. No. 110189). Collagen type I was crosslinked to the PAA gel surface using sulfo‐SANPAH (Proteochem, Hurricane, UT, USA, Cat. No. 102568‐43‐4). The gels were submerged under 1 mg·mL^−1^ sulfo‐SANPAH solution and placed 2 inches below an 8 W ultraviolet UV lamp (Hitachi F8T5 – 365 nm, Chiyoda City, Tokyo, Japan) and irradiated for 15 min. The gels were then washed with HEPES buffer and soaked with 0.1 mg·mL^−1^ rat‐tail collagen type I (Corning, Corning, NY, USA, Cat. No. 354249) for 12 h at 4 °C. After collagen coating, the collagen was aspirated, and gels were placed in culture medium and incubated for 30 min at 37 °C before cells were seeded on them.

For traction force microscopy, we followed an established protocol [62] to fabricate gel disks of 18 mm in diameter and approximately 100 μm in thickness. These gel disks were prepared with 0.1 μm red fluorescent beads (Life Technologies, Carlsbad, CA, USA, Cat. No. F8801) embedded just beneath the top surface. 25 mm × 25 mm square glass coverslips (VWR, Atlanta, GA, USA, Cat. No. 48368‐085) were cleaned, treated with 1% 3‐aminopropyl‐trimethoxysilane (Sigma‐Aldrich, CAS# 13822‐56‐5) solution for 10 min, and then coated with 0.5% glutaraldehyde. Round glass coverslips (VWR, Cat. No. 48382‐042), 18 mm in diameter, were plasma cleaned and coated with a thin layer of fluorescent beads. A 25 μL mixture of acrylamide, bis‐acrylamide, and initiators was applied between the glutaraldehyde‐coated square coverslip and the beads‐coated round coverslip, followed by polymerization at room temperature for 15 min. Subsequently, the round coverslip was gently removed, leaving the resulting gel disk firmly attached to the square coverslip, with the embedded beads positioned within 2 μm below the gel surface. To allow for the increased cell numbers needed for RNA testing, PAA gels were prepared onto 75 × 51 mm microscope slides (Electron Microscopy Sciences, CAS# 71862–01) according to previously described protocol [63].

### Lentivirus preparation and infection

HEK‐293T cells were maintained at 37 °C in Dulbecco's modified Eagle medium (DMEM), supplemented with 10% FCS and antibiotics (100 units·mL^−1^ penicillin and 100 μg·mL^−1^ streptomycin). Cells were transfected with pVSV‐G [64] and pCMV∆R8.91 [65], together with the pLKO.1‐puro non‐targeting vector (Sigma Mission clone SHC016; ‘shCTRL’) or the pLKO.1‐shRNA vector (Sigma Mission TRCN0000001418; ‘shDDR2’) using Lipofectamine™ 2000 reagent (ThermoFisher Scientific, Cat. No. 11668027) as recommended by the manufacturer and following the recommendations of the RNAi Consortium (TRC) laboratory protocols with slight modifications. Twelve hours after transfection the medium was replaced by DMEM, supplemented with 30% FCS and antibiotics which. Cell supernatants were harvested every 24 h, replaced with fresh medium, and stored at 4 °C until collection of the last harvest (at 72 h). At this point, the consecutive harvests were pooled, filtered through 0.45 mm filters and split into 3–5 mL aliquots, which were stored at −80 °C. SH‐SY5Y cells were infected with shCTRL or shDDR2 lentiviral particles by adding a 1:1 mix of medium:viral supernatant for 24–48 h. Puromycin selection (2 μg·mL^−1^) was applied for 2–3 days and always compared to non‐transduced control cells, which generally died within the first 24 hs. DDR2 downregulation was confirmed by qPCR.

### RNA sequencing and data analysis

Total RNA was extracted using TRIzol Reagent (ThermoFisher Scientific, Cat. No. 15596026) followed by RNA integrity analysis by Fragment Analyzer HS RNA assay (Agilent Technologies, Santa Clara, CA, USA, Cat. No. DNF‐472‐0500) and quantification by Qubit RNA HS assay (ThermoFisher Scientific, Cat. No. Q32855). Strand‐specific total RNA‐Seq libraries were prepared using NEBNext rRNA Depletion Kit v2 (Human/Mouse/Rat) kit (NEB, Ipswitch, MA, USA, Cat. No. E7400L), NEBNext Ultra™ II Directional RNA Library Prep kits (NEB, Cat. No. E7765L) and IDT xGEN UDI primers (Integrated DNA Technologies, Coralville, IA, USA, Cat. No. 10008052) according to manufacturers' instructions. The final total RNA‐Seq libraries were quantified by Fragment Analyzer HS NGS assay (Agilent Technologies, Cat. No. DNF‐474‐0500) and Qubit dsDNA HS assay (ThermoFisher Scientific, Cat. No. Q32854), multiplexed and sequenced by paired‐end 150 bp (PE150) using NovaSeq 6000 SP v1.5 (300 cycles) kit (Illumina, San Diego, CA, USA, Cat. No. 20028400). Reads were sorted by the barcodes assigned to each library and adapter sequences were removed using trimmomatic [66]. The reads were then mapped to the human genome through star [67], and the gene expressions count table was generated by tetranscripts [68]. DEGs were identified using an r package deseq2 [69]. GO analysis was performed using another r package clusterprofiler [70].

### RT‐quantitative PCR

RNA was extracted as described above and 1200 ng of RNA was reverse transcribed into cDNA using a SuperScript IV First‐Strand Synthesis System (ThermoFisher Scientific, Cat. No. 18091050) using Oligo d(T)_20_ following the kit's manual. Reverse transcription‐quantitative PCR was performed in three to six biological repeats in 96‐well plates using Azure Cielo 6 Real‐Time PCR system (part #: AIQ060) with Applied Biosystems Power SYBR Green PCR Master Mix (ThermoFisher Scientific, Cat. No. 4368706). 600 nm of forward and reverse primers, and 100–125 ng of cDNA were used in each 25‐uL reaction. The cycling conditions were as recommended in the manufacturer's guide. All transcript levels were normalized to the GAPDH transcript level. Primers used in this study was included in Table S6.

### Cell senescence analysis

Cells were plated onto collagen‐coated PAA gels of desired stiffness and allowed to incubate for 48 h. The medium was removed from cells after 24 h. Cells were rinsed with pre‐warmed 1x PBS and fixed with 1× fixative solution provided by senescence beta‐galactosidase staining kit (Cell Signaling Technology, Danvers, MA, USA, Cat. No. 9860) for 15 min. Fresh beta‐galactosidase staining solution was prepared according to manufacturer's instructions and pH was confirmed to be 6.0. Cells are washed 2× with 1× PBS and 3 mL of staining solution are added to each dish. Dishes were wrapped in parafilm to avoid evaporation and incubated at 37 °C in a dry incubator until blue color was visible (48 h). The beta‐galactosidase positive cells (blue) were considered as positive senescent cells.

### Cell proliferation assay

Cell proliferation was assessed using, ethynyl‐2′‐deoxyuridine (EdU) incorporation using Click‐iT EdU imaging kit (ThermoFisher Scientific, Cat. No. C10337). Cells were seeded onto collagen‐coated PAA gels of varying stiffnesses for 24 h. Following overnight incubation and relaxation of cells, half of the cell media was removed and replaced with 20 μm EdU working solution and allowed to incubate in ideal culture conditions for 4 h. After the 4 h of EdU labeling, cells are fixed using 3.7% formaldehyde in PBS for 15 min at room temperature. After fixation, cells are rinsed 2× with 3% BSA in PBS. The washing solution was removed, and cell membranes were permeabilized with 0.5% Triton‐X in PBS incubated at room temperature for 20 min. Permeabilization solution was removed, and dishes are rinsed 2× with 3% BSA in PBS. The reaction cocktail was prepared fresh and according to the manufacturer's protocol and incubated on each dish at room temperature for 30 min, protected from light. The reaction cocktail is removed, and dishes are washed once with 3% BSA in PBS. Hoechst 33342 (ThermoFisher Scientific, Cat. No. H1399) is diluted at 1 : 2000 in PBS and incubated in each dish for 30 min at room temperature, protected from light. Dishes were rinsed 2× with PBS. The final dishes were imaged in PBS. EdU and Hoechst 33342 signals were captured in separate channels using a Zeiss LSM 700 confocal microscope (White Plains, NY, USA) with a water immersion 20× 1.0 NA objective, and maximum projection images were processed with imagej software (NIH, Maryland, MD, USA). Microscopy settings were held constant between experiments. Green EdU signal was considered as a positive EdU signal.

### Phalloidin imaging

Cells were incubated onto gels for 24 h; pre‐warmed PBS was used to rinse cells 3 times. After washing, cells were immediately fixed using a 3.7% formaldehyde for 15 min at room temperature. Cells are washed twice with PBS, and cell membranes are permeabilized using a 0.1% Triton‐X solution for 20 min. After Triton‐X incubation, cells are washed 2 times with PBS. To stain F‐Actin within cells, 0.5 μL of 400× stock of Phalloidin Alexa 488 nm (ThermoFisher Scientific, Cat. No. A12379) is added per 200 μL of PBS to each sample and incubated at room temperature away from light for 1 h. Cells are then washed 2× PBS. Final dishes were imaged in PBS under Zeiss LSM 700 confocal microscope at 20× 1.0 NA and Z‐stacks were taken from the bottom to top of individual cells. Microscopy settings were held constant between experiments. Average projections of z‐stacks were created within imagej.

### Cellular spreading area measurements

Cells were seeded with 20% confluency on PAA gels for 24 h. Individual cells were imaged using an Olympus IX83 inverted microscope equipped with a 40× 0.6 NA objective using the Phase contrast mode. Cell area was measured from the phase contrast images using fiji imagej software (NIH). Cell boundaries were traced using the free‐hand tool and the area enclosed by the cell boundary was measured as cell area.

### Cell traction force measurements

Cell traction forces were measured using traction force microscopy [62]. Cells were cultured on PAA substrates for 24 h before subjected to traction force microscopy. For each cell selected for traction force microscopy, a fluorescence image of the substrate was recorded to capture the marker beads in the stressed state. In addition, a phase contrast image was acquired to record the morphology of the cell. Trypsin (ThermoFisher Scientific, Cat. No. 25200056) was then applied to disrupt cell‐substrate interactions and cause the cell to detach from the substrate. A final fluorescent image of the substrate was taken to capture the marker beads in the relaxed, unstressed state. Bead displacements were calculated from the two fluorescent images using a particle image velocimetry toolbox written in matlab [71]. Traction stress on the gel surface was calculated from the bead displacements using the finite element analysis software (Ansys, Inc., Canonsburg, PA, USA). The magnitude of total traction force (F) was calculated by integrating the magnitude of traction stress over the cell area.

## Conflict of interest

The authors declare no conflict of interest.

### Peer review

The peer review history for this article is available at https://www.webofscience.com/api/gateway/wos/peer‐review/10.1002/2211‐5463.13798.

## Author contributions

TV, SX, MS, QW and HSZ conceived and designed the experiments. TV, SX and MS prepared the experimental materials. TV, SX, MS, JF, and ER performed the experiments. TV, SX, MS, JF, CX, ER, QW and HSZ interpreted and analyzed the data. TV, SX, CX, ER, HSZ and QW wrote the manuscript. ER and JS provided the cell lines.

## Supporting information


**Fig. S1.** DDR2 downregulation by shRNA treatment. A) RNA‐seq showing reduction of DDR2 on the gene level. B) Reduction of the DDR2 mRNA level validated by q‐PCR.
**Fig. S2.** The normalized sequencing reads counts of genes involved in cell cycle and cellular senescence pathways from shCTRL vs shDDR2 cell lines. (A) The normalized reads count of *MYBL2*, *BUB1*, *PLK1*, *CCNE1* and *CCNB1* in shCTRL vs shiDDR2 cell lines. (B) The normalized reads count of *PTEN*, *NF2* and *TGF*b*1* in shCTRL vs shDDR2 cell lines. (C) The normalized reads count of *TERT, CDKN1A* and *IGF2* in shCTRL vs shDDR2 cell lines.
**Fig. S3.** qPCR analysis of shCTRL and shDDR2 cell lines. *, p<0.05, student t‐test.
**Fig. S4.** Representative phase contrast images of cellular morphology of shCTRL and shDDR2 on both 2 kPa and 20 kPa PAA gels. Scale bars represent 10 *μ*m.

**Table S1.** Stiffnesses of PAA Gel.

**Table S2.** Differentially expressed genes in shDDR2 versus shCTRL cells cultured in 2 kPa substrate.

**Table S3.** Differentially expressed genes in shDDR2 versus shCTRL cells cultured in 20 kPa substrate.

**Table S4.** Gene ontology analysis of the biological process of those downregulated DEGs in shDDR2 versus shCTRL cells cultured in 2 kPa substrate.

**Table S5.** Gene ontology analysis of the biological process of those upregulated DEGs in shDDR2 versus shCTRL cells cultured in 2 kPa substrate.

**Table S6.** List of primers used in qPCR experiments.

## Data Availability

RNA‐seq data have been deposited at GEO with the accession number GSE246550 and will be publicly available upon publication. All other data are available upon request to the corresponding author Dr. Qi Wen (qwen@wpi.edu).
