# Supplementary material for: DDR2 signaling and mechanosensing orchestrate neuroblastoma cell fate through different transcriptome mechanisms
Source: FEBS Open Bio. 2024 Mar 27;14(5):867–82. doi: 10.1002/2211-5463.13798 (PMC11073507; doi:10.1002/2211-5463.13798)
Supplement: Supplementary file 1 — Fig. S1. DDR2 downregulation by shRNA treatment. A) RNA‐seq showing reduction of DDR2 on the gene level. B) Reduction of the DDR2 mRNA level validated by q‐PCR. Fig. S2. The normalized sequencing reads counts of genes involved in cell cycle and cellular senescence pathways from shCTRL vs shDDR2 cell lines. (A) The normalized reads count of MYBL2, BUB1, PLK1, CCNE1 and CCNB1 in shCTRL vs shiDDR2 cell lines. (B) The normalized reads count of PTEN, NF2 and TGFb1 in shCTRL vs shDDR2 cell lines. (C) The normalized reads count of TERT, CDKN1A and IGF2 in shCTRL vs shDDR2 cell lines. Fig. S3. qPCR analysis of shCTRL and shDDR2 cell lines. *, p<0.05, student t‐test. Fig. S4. Representative phase contrast images of cellular morphology of shCTRL and shDDR2 on both 2 kPa and 20 kPa PAA gels. Scale bars represent 10 μm. [file FEB4-14-867-s004.pdf]

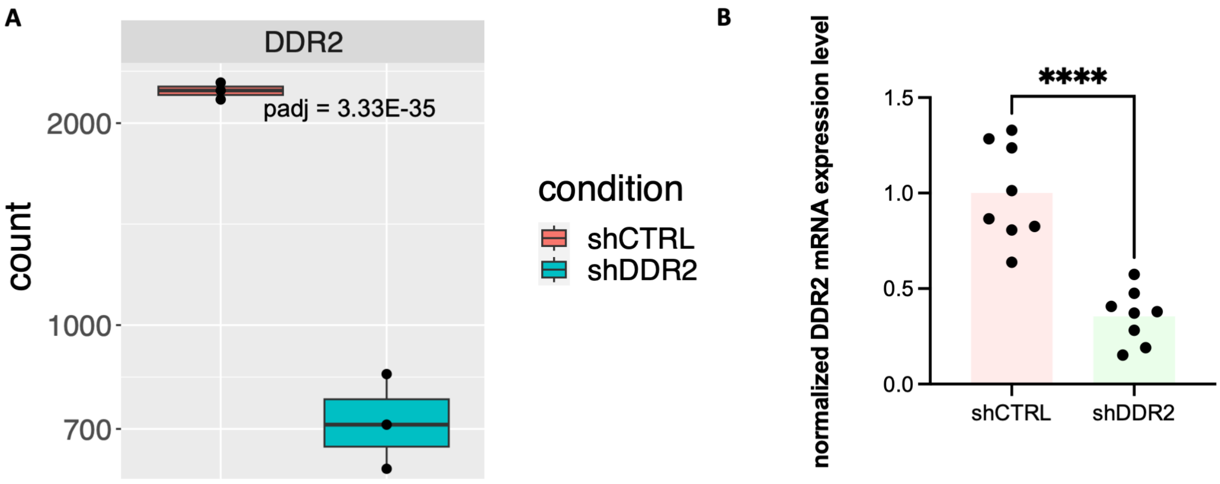


**Figure S1.** DDR2 downregulation by shRNA treatment. A) RNA-seq showing reduction of DDR2 on the gene level. B) Reduction of the DDR2 mRNA level validated by q-PCR .

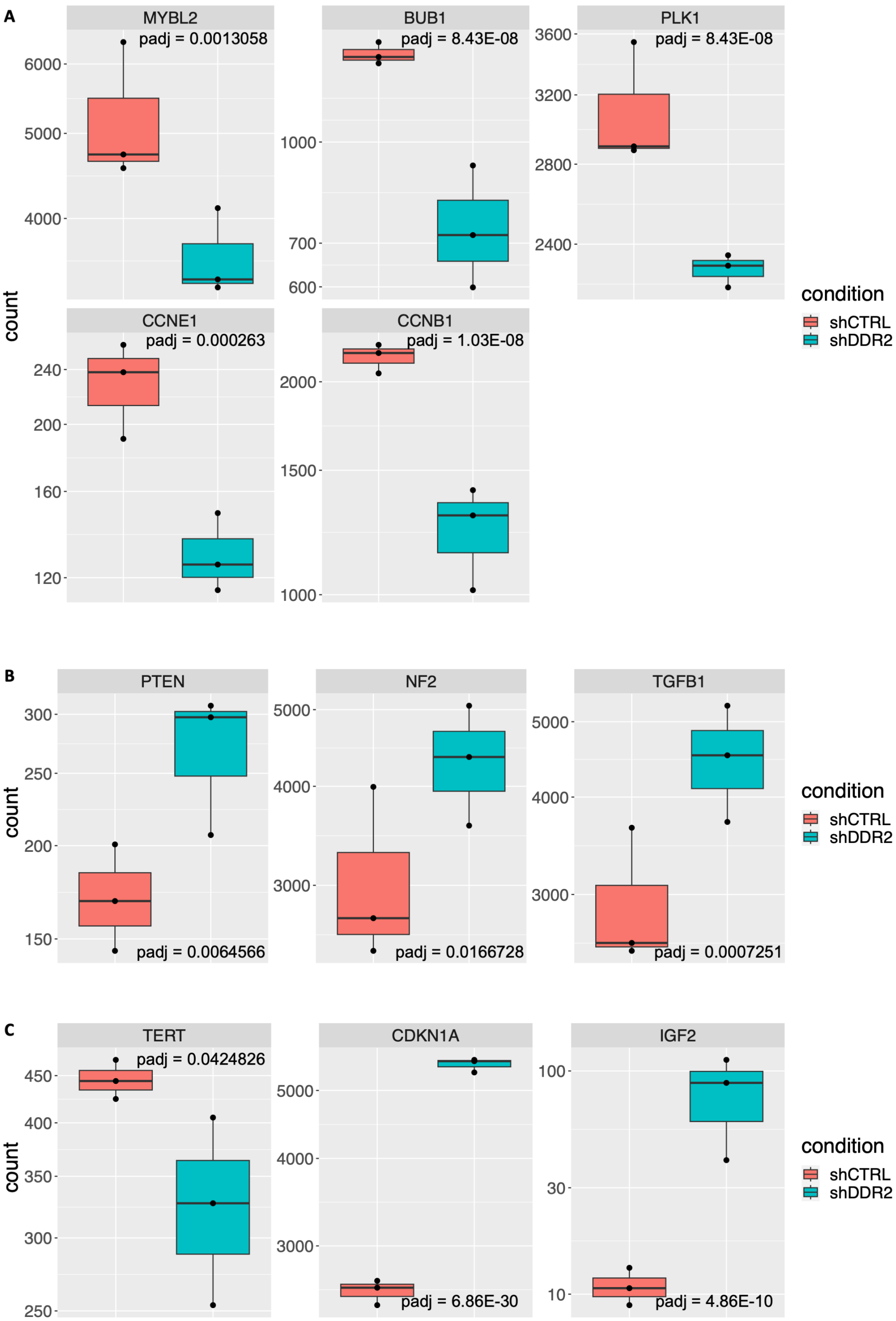


**Figure S2.** The normalized sequencing reads counts of genes involved in cell cycle and cellular senescence pathways from shCTRL vs shDDR2 cell lines. (A) The normalized reads count of *MYBL2*, *BUB1*, *PLK1*, *CCNE1* and *CCNB1* in shCTRL vs shiDDR2 cell lines. (B) The normalized reads count of *PTEN*, *NF2* and *TGFβ1* in shCTRL vs shDDR2 cell lines. (C) The normalized reads count of *TERT, CDKN1A* and *IGF2* in shCTRL vs shDDR2 cell lines.

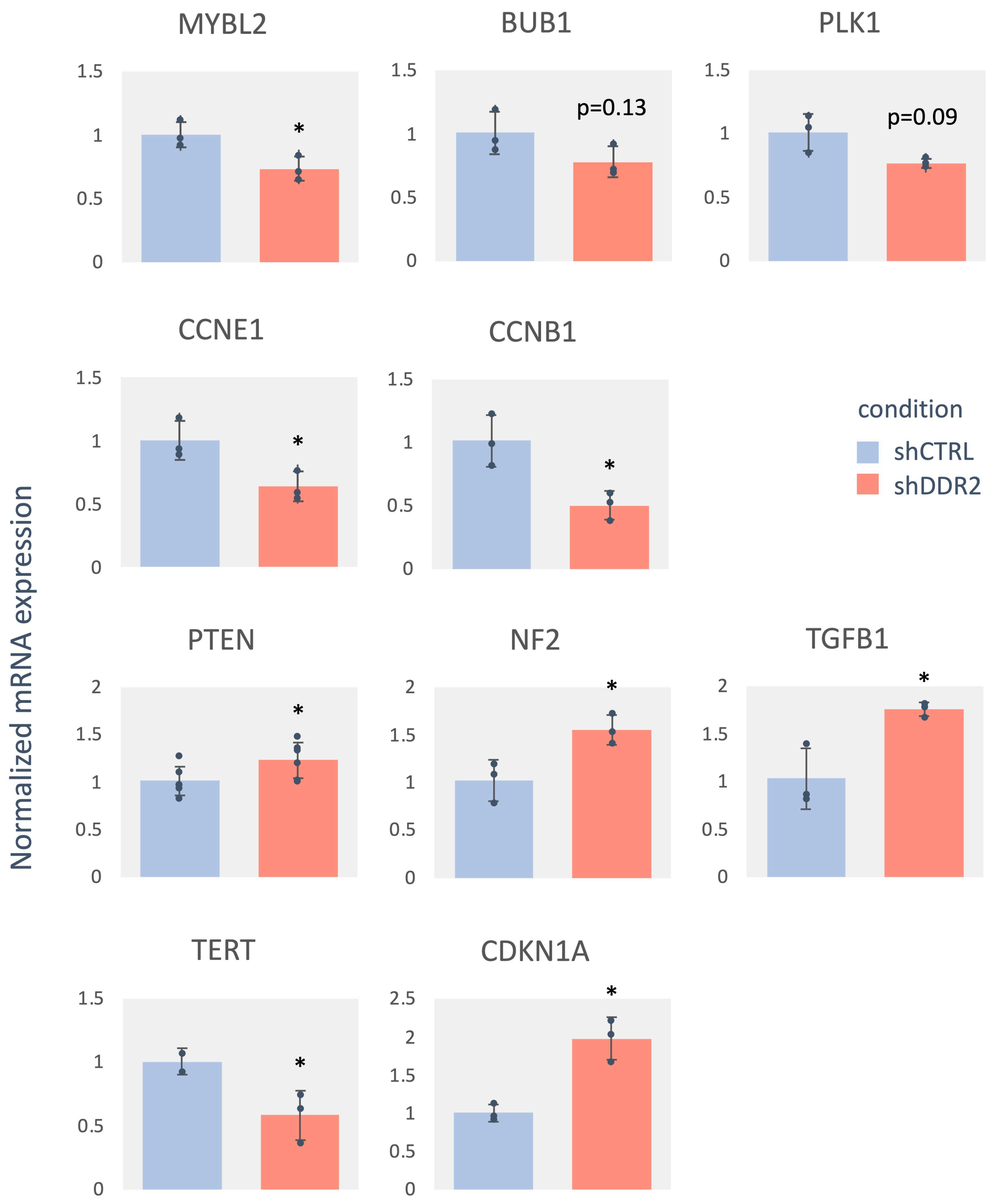


**Figure S3.** q-PCR analysis of shCTRL and shDDR2 cell lines. *, $p<0.05$, student t-test.

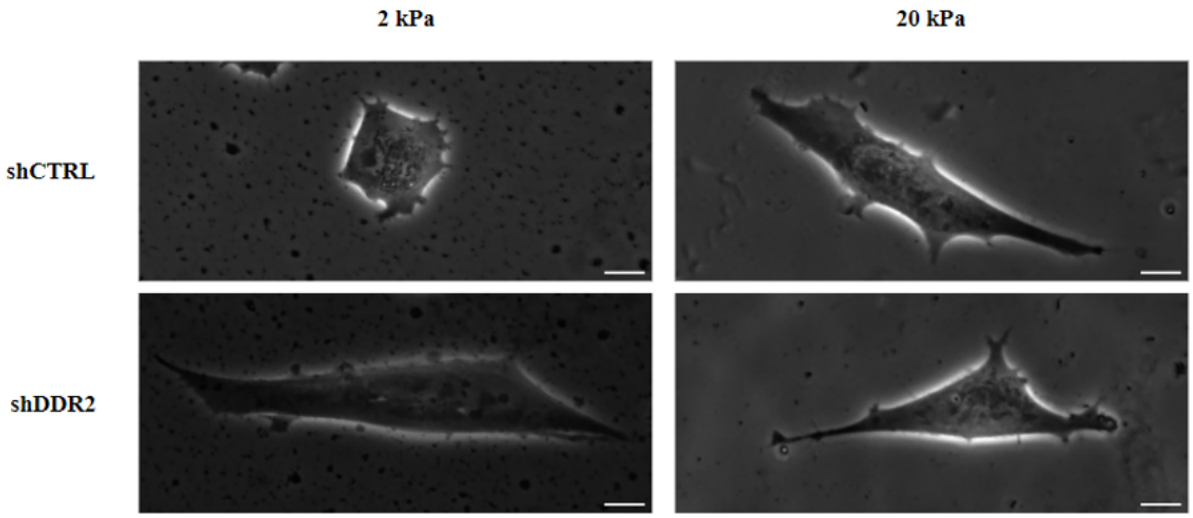


**Figure S4.** Representative phase contrast images of cellular morphology of shCTRL and shDDR2 on both 2 kPa and 20 kPa PAA gels. Scale bars represent 10 $\mu$m.
