# Supplementary material for: DDR2 signaling and mechanosensing orchestrate neuroblastoma cell fate through different transcriptome mechanisms
Source: FEBS Open Bio. 2024 Mar 27;14(5):867–82. doi: 10.1002/2211-5463.13798 (PMC11073507; doi:10.1002/2211-5463.13798)
Supplement: Supplementary file 2 — Table S1. Stiffnesses of PAA Gel. [file FEB4-14-867-s006.pdf]

Table S1. Stiffnesses of PAA Gels

Stiffness (kPa)	Acrylamide (%)	Bis acrylamide (%)
0.8	2	0.12
2	5	0.08
7.5	8	0.08
13	10	0.12
20	12	0.14
